# Giant Hemorrhagic Pancreatic Pseudocyst: A Case Report and Guidelines for Care

**DOI:** 10.7759/cureus.28398

**Published:** 2022-08-25

**Authors:** Simon Sabir, Sapphire Peace, Crystal Ho, Kyle Oi, Khoa Le

**Affiliations:** 1 Internal Medicine, Touro University Nevada, Henderson, USA; 2 Internal Medicine, Universidad Autónoma de Guadalajara, Guadalajara, MEX; 3 Internal Medicine, North Vista Hospital, Las Vegas, USA

**Keywords:** pancreatic pseudocyst, acute pancreatitis, alcohol, pseudocyst, giant, hemorrhagic, chronic pancreatitis

## Abstract

Pancreatic pseudocysts are potential sequelae of acute or chronic pancreatitis. In some cases, enzymatic degradation of the lining between a pseudocyst and the splenic artery, or surrounding vessels, can occur, resulting in a hemorrhagic pancreatic pseudocyst. Very few of these hemorrhagic pseudocysts meet the criteria for giant pseudocysts. We discuss the case of a 30-year-old male patient with a history of alcohol abuse who presented to the hospital with a giant hemorrhagic pancreatic pseudocyst; he was admitted for expectant management and was subsequently discharged. This case report seeks to shed light on the dearth of similar cases.

## Introduction

Pancreatic pseudocysts are a complication of either acute or chronic pancreatitis, and they occur in about 15% of patients with acute pancreatitis and 30-40% of patients with chronic pancreatitis [1,2]. They are often asymptomatic, painless abdominal collections; however, they can present clinically as a result of mass effect: abdominal pain, weight loss, non-bilious vomiting, and early satiety, as well as with symptoms of biliary obstruction. They can also have more catastrophic outcomes, such as cyst ruptures, infection, and hemorrhage secondary to ruptured hemorrhagic cysts or arterial bleeding [2]. Ultimately, the management is based on the size and type of pseudocyst, maturity of the cyst wall, and location, with consideration for the proximity of the cyst relative to adjacent structures. Existing treatment modalities and proposed modalities are discussed later in this paper. We discuss the presentation and treatment of a 30-year-old male who presented to the hospital with a giant hemorrhagic pancreatic pseudocyst.

Pancreatic pseudocysts are thought to result from insults to the pancreatic duct due to either pancreatitis or another trauma. The pathogenesis varies depending on whether the cause is acute or chronic pancreatitis. In acute pancreatitis, the pseudocyst formation and enlargement are related to the accumulation of enzyme-rich pancreatic juices and products of auto-degradation. In chronic pancreatitis, the pseudocyst formation is associated more with duct obstruction and lateral dilatation of structures [3]. In either case, the pseudocyst is now encapsulated within fibrous or granulomatous tissue. Depending on the size of the pseudocyst, and whether or not it communicates with a pancreatic duct, the pseudocyst may either increase, decrease, or stagnate in size. It is this size that will usually determine the clinical presentations, notwithstanding any complications related to rupture or bleeding [2].

The progression of pancreatitis to pancreatitis with hemorrhage due to vascular complications represents a devastating turn of events, resulting in a patient mortality rate between 34-52% [4]. Clinically, the patients are often asymptomatic until hemorrhage has occurred, with the bleed followed by hemodynamic instability and a decline in hematocrit [5]. Immediate treatment is needed to optimize the likelihood of patient survival.

Development of intrapancreatic bleeds occurs due to processes that result in the disruption of the surrounding vasculature including the release of pancreatic enzymes that lead to local cytokine activation and digestion of vasculature. A variety of bleeds can occur in patients with pancreatitis. Of note, 60% of these complications occur due to the rupture of a pseudoaneurysm while 20% of bleeds occur due to small vessel hemorrhage or hemorrhage without pseudoaneurysm; the remaining 20% of bleeds are caused by hemorrhagic pseudocysts without pseudoaneurysms [5].

Finally, pancreatic pseudocysts are described in the literature, based on their sizes, ranging from those as small as <2 cm, medium-sized (2-6 cm), large (>6 cm), to giant ones (≥10 cm) based on the major diameter [6].

## Case presentation

**Figure 1:**
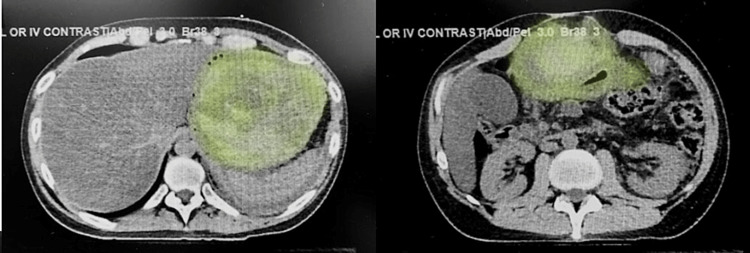
Contrast-enhanced CT scan of the abdomen: axial view The images show a large pancreatic pseudocyst (green overlay) occupying almost the entire left half of the abdomen and protruding anteriorly CT: computed tomography

A 30-year-old male who presented with epigastric abdominal pain was admitted to the ED for evaluation. He was known to have a remote history of a single episode of pancreatitis one year ago and had been treated at a different hospital with supportive measures. The patient reported that he had stopped drinking alcohol two weeks prior to the onset of abdominal pain. Physical exam was significant for epigastric tenderness and a tense, distended abdomen. CT scan showed hemorrhagic pancreatitis with the formation of a giant heterogeneous hematoma involving the pancreatic body and tail measuring 16.2 x 12.8 x 10.8 cm causing significant anterior compression and displacement of the stomach (Figure 1). Laboratory data are presented in Table 1.

**Table 1:** Laboratory values

Labs	Reference Range (Male)	Patient Values
Hemoglobin	12–16 g/dL	9.0 g/dL
Hematocrit	42–50%	27.4%
Mean Corpuscular Volume	80–98 μm^3^	103.7 μm^3^
Leukocyte Count	4,000–11,000/µL	14,800/µL
C-Reactive Protein	≤0.8 mg/dL	26.1 mg/dL
Alanine Aminotransferase	10–40 IU/L	47 IU/L
Aspartate Aminotransferase	10–40 IU/L	83 IU/L
Alkaline Phosphatase	30–120 IU/L	104 IU/L
Total Bilirubin	0.3–1 mg/dL	0.6 mg/dL
Serum Amylase	80–180 units/dL	43 units/dL
Serum Lipase	10–140 units/L	354 units/L
Blood Urea Nitrogen	8–20 mg/dL	8.0 mg/dL
Serum Creatinine	0.70–1.30 mg/dL	0.5 mg/dL

## Discussion

Existing workup

The American College of Gastroenterology released guidelines on the evaluation of pancreatic cysts in 2018 and offered strengths of recommendation for those guidelines. MRI or MR cholangiopancreatography (MRCP) are the imaging modalities of choice when confirming the diagnosis of a pancreatic pseudocyst, and this usually follows indications of an encapsulated fluid mass on ultrasound (US). CT or US can be alternatives for patients unable to undergo MRIs. Finally, indeterminate cysts may receive more than one type of imaging, and they may receive endoscopic US with fine needle aspiration (EUS with FNA) for cyst fluid analysis [7].

Serum tests may provide some insight into the underlying etiology of pancreatitis. However, they have limited utility for diagnosing pancreatic pseudocysts; amylase, lipase, coagulation studies, bilirubin, and transaminases may assist in trending changes if they deviate from normal [2]. This case report shows that serum labs did not offer much utility or indication of a pseudocyst.

Management options described in the literature involve one of the three following general principles: drainage of the pseudocyst, thrombosis of involved vessels, or expectant management.

Here we describe the technique, outcomes, prognosis, and potential complications of drainage and hemostatic methods.

Endoscopic drainage

Beginning with endoscopic drainage (ED), there are two approaches we have reviewed. ED of a pseudocyst is done either via a transpapillary or transmural route, and the latter is considered a cystogastrostomy (CG). EUS is used to determine the pseudocyst's communications and location. A transpapillary pseudocyst drainage uses endoscopic retrograde cholangiopancreatography (ERCP) guidance to place a stent through the ampulla of Vater and into the pancreatic duct, assuming there is communication between the duct and the pseudocyst [8]. Regarding the success of stent placement and resolution of pancreatic duct obstructions, a 2005 review of 95 patients with stents placed showed a long-term resolution of pancreatic duct placement in 94% of patients who had a successful initial resolution of duct obstruction [9]. Success depended on the length and location of the stent, as well as the degree of pancreatic duct obstruction (partial vs. complete); there were higher rates of resolution in stenting of partial duct obstructions (88%) versus complete duct obstruction (12%). Complications in the aforementioned review included a case of fatal multisystem organ failure and cases of stent occlusion (7.1%) [9].

Cystogastrostomy

CG is transmural drainage that uses endoscopy with EUS guidance to locate, confirm, puncture, stent, and drain a pseudocyst that is directly in contact with either the gastric or duodenal wall [8]. This approach is the current mainstay of therapy in the treatment of pancreatic pseudocysts. However, the data on its use for giant hemorrhagic pancreatic pseudocysts is sparse. It is a preferred method when there is communication between the pseudocyst and the stomach, as it can be done endoscopically and concurrent biopsies can be taken to screen for malignancy, such as pancreatic adenocarcinoma. A retrospective study at the West China Hospital of Sichuan University compared CG with Roux-en-Y cystojejunostomy (RCJ) and concluded that there was no significant difference (p=0.467) in recurrence rates between the two methods of cyst drainage. Ultimately, there was a 7.5% recurrence rate in the CG cohort assessed (n=119). Compared to RCJ’s 22.5% complication rate, CG had significantly fewer complications at 10.9%. Additionally, CG was associated with significantly less operating time, gastric tube retention time, and total expenses compared to RCJ [10].

Percutaneous drainage

Percutaneous drainage (PD) involves either simple percutaneous aspiration or catheter placement and is guided by CT or US [11]. The use of PD as a form of noninvasive pseudocyst management has fallen out of favor in recent years. The outcomes of noninvasive drainage techniques were compared in one study involving 164 patients: 55 of whom had PD while 109 underwent ED. In the 109 patients who underwent ED, treatment success was considerably higher compared to those managed by PD (70% vs. 31%), with success being defined as the “complete resolution or a decrease in the size of the pancreatic fluid collections (PFC) to less than 2 cm on follow-up CT” [12]. Additionally, ED required fewer interventions (median of 1.8% vs. 3.3%). It should be noted that no patient in the study was found to have a hemorrhagic pseudocyst.

Roux-en-Y cystojejunostomy

RCJ is a laparoscopic technique that creates an anastomosis between the proximal Roux end of the jejunum (created after dividing the jejunum ~25-30 cm distal to the gastroduodenal junction) and the most dependent part of the pseudocyst. This can be accessed either through the transverse mesocolon or the gastrocolic omentum [13]. Finally, an entero-entero anastomosis is created to allow for gastric contents to drain into the jejunum about 40 cm distal to the Roux end.

Laparoscopic cystogastrostomy

Laparoscopic cystogastrostomy typically involves accessing the pseudocyst through the posterior aspect of the gastric compartment. Indications for the procedure are symptomatic cysts or patients experiencing complications secondary to cyst formation [14]. With regard to complications secondary to the procedure, one analysis looked at the different post-surgical complications in 347 patients who underwent PD and 248 patients who underwent laparoscopic drainage (LD). The rates of intraabdominal abscess were 9.8% in PD vs. 5.6% in LD; the sepsis rates were 31.4% in PD and 13.7% in LD, and respiratory failure rates were 17.3% in PD and 9.7% in LD [15]. Within these groups, laparoscopic drainage was selected as the most successful approach.

Endovascular coiling

Finally, regarding thrombotic methods, hemorrhagic pseudocysts are formed when enzymes within pancreatic fluid erode arterial walls, and the most commonly affected artery is the splenic artery (30-50%), followed by gastroduodenal (17%), and pancreaticoduodenal arteries (11%) [16]. Angiographic embolization has a success rate ranging from 79 to 100% [17].

Coils are the most widely used tool for embolization. They act by introducing a mechanical obstruction that slows the speed at which blood flows through the vasculature. Additionally, they induce thrombosis via their thrombogenic fibers and by inciting inflammatory reactions. The main aim of coil embolization is the occlusion of the pseudoaneurysm. Pseudoaneurysms that occur within the visceral vasculature, including in the splenic, hepatic, and gastroduodenal arteries, require embolization through the sandwich technique. This technique is commonly used in vasculature with collateral circulation, and it has clinical success rates of >90%. With this technique, the occlusion is done distal; the efferent artery is done first, followed by the occlusion of the afferent, proximal artery. Embolization of only the afferent artery will lead to recurrence due to retrograde filling from the efferent collateral [18,19]. 

In one study involving 76 patients, three patients died, two from hemorrhagic shock and one from brain stem infarction secondary to coagulation system dysfunction in severe acute pancreatitis [17].

Hemostasis using factor VII

The initiation of coagulation begins with the formation of rFVIIa and tissue factor complex released at the point of vessel wall injury where it imposes its prothrombotic effect by activating factors X and XI in the coagulation cascade. Factor VII acts exclusively at the site of endothelial damage, and this lack of systemic activation has proven highly useful to its implementation as a hemostatic agent. Currently, the agent bears no known link to disseminated intravascular coagulation or thromboembolic events. Promising data has been released regarding the use of rFVII in intra-abdominal bleeds. This therapy has shown the potential to arrest growth in hemorrhagic pseudocyst size, further preventing secondary complications related to mass effect and cyst rupture including hemoperitoneum, shock, and peritonitis. To address the concern of thromboembolic events, von Heyman et al. performed an analysis of eight publications that described placebo-controlled studies on rFVIIa therapy in connection with surgical interventions; they described a total of 285 patients receiving placebo and 555 patients who received rFVIIa as a prophylactic measure to reduce the risk of hemorrhage. Based on their findings, the mean estimated risk of thromboembolism was 5.97% for placebo compared with 6.42% for rFVIIa [20].

## Conclusions

Giant hemorrhagic pancreatic pseudocysts have not been directly reviewed as there is limited literature, if any, on their occurrence. Ultimately, expectant management was successful in the treatment of our patient, suggesting that it is an option to consider in hemodynamically stable patients whose symptoms are not refractory to conservative treatment. However, after careful review, we do propose some therapeutic approaches to giant pancreatic pseudocysts and hemorrhagic pancreatic pseudocysts. Using pseudocyst recurrence rates and complication rates as measures of success in pseudocyst drainage techniques, laparoscopic CG has the lowest of both rates when compared to PD and endoscopic CG; this makes laparoscopic CG a successful approach and method to manage pseudocysts, especially those that have favorable proximity between the pseudocyst and gastric wall. It has also been associated with significantly lower mean inpatient length of stay and total costs. Regarding thrombotic methods, endovascular coiling has proven to be a highly successful and safe method to treat pancreatic hemorrhage. The existing studies describing factor VII treatment recognize its promise as a future adjunct to existing hemostatic treatment.
